# Failure of Ketamine Anesthesia in a Patient with Lamotrigine Overdose

**DOI:** 10.1155/2014/916360

**Published:** 2014-07-09

**Authors:** Daniel Kornhall, Erik Waage Nielsen

**Affiliations:** ^1^Department of Anesthesiology, Nordland Hospital, 8095 Bodø, Norway; ^2^Institute of Clinical Medicine, University of Tromsø, 9037 Tromsø, Norway; ^3^University of Nordland, 8049 Bodø, Norway

## Abstract

*Introduction.* It is important to know which clinical situations prevent ketamine from working.* Case Report. *We present the case of the psychiatric inpatient who was admitted to our emergency department after ingesting a toxic dose of lamotrigine, unknown at that time. On admission, she was clearly in distress, displaying extreme agitation and violent ataxic movements. We opted to achieve sedation using intravenous ketamine boluses. Unexpectedly, after being injected with a total of 250 mg ketamine, our patient displayed no signs of dissociative anaesthesia.* Discussion. *There was no apparent reason for why ketamine failed, but an interaction between lamotrigine and ketamine was suspected. A literature search was performed. Very few articles describe interactions between lamotrigine and ketamine. Experimental studies, however, demonstrate how lamotrigine attenuates the neuropsychiatric effects of ketamine. Ketamine is classically described as an NMDA antagonist. Ketamine's dissociative effects, however, are thought to be mediated by increased glutamate release via a pathway not dependent on NMDA receptors. Lamotrigine, on the other hand, is known to reduce cortical glutamate release.* Conclusion. *Lamotrigine reduces the glutamate release needed to mediate ketamine's dissociative anaesthesia. This is important knowledge for anaesthesiologists in the emergency room where ketamine is often administered to unstable patients.

## 1. Introduction

Ketamine is an effective agent for procedural sedation in the emergency department. It is generally considered a safe anaesthetic agent as it, in most patients, preserves hemodynamics and respiratory drive.

## 2. Case Presentation

Our local ambulance service was dispatched to a young woman with generalised seizures. The patient was transported severely agitated to our emergency department. The emergency department staff requested the assistance of the on-call anaesthetist and a nurse anaesthetist to sedate the patient. At that time, several nurses and orderlies had to manually restrain the patient to prevent further harm as she displayed violent, bizarre, ataxic movement that appeared consciously initiated but very poorly controlled. Nystagmus was also noted.

In her agitated state, no meaningful monitoring, examination, or assessment was possible. Obtaining blood samples and a CT-scan was impossible. We decided to sedate the patient with ketamine. For the majority of patients, sedation is achieved with intravenous doses in the range 0.5–1.0 mg/kg [1]. We estimated our patient's weight to be 80 kg.

While manually restraining the patient, two large bore antecubital intravenous lines were established and tested with saline injections. The blood pressure was 113/87 mmHg and the pulse was 103 bpm. The patient was oxygenated using a nonrebreather mask. Ketamine was injected in boluses of 50 mg before waiting for 2-3 minutes while observing signs of dissociative sedation. Over approximately ten minutes, a total ketamine dose of 100 mg was injected in the left antecubital cannula without any signs of dissociative anaesthesia. During another 10 minutes, another 150 mg of ketamine was injected via the contralateral cannula. Both cannulae were patent. After 20 minutes, our patient had been injected with a total ketamine dose of 250 mg without displaying any signs of sedation. Ataxic movements and agitation had possibly increased in intensity. The patient appeared euphoric.

At that stage, a rapid sequence induction with 120 mg propofol and 80 mg succinylcholine was performed. Our patient promptly became comatose and was uneventfully intubated. A thorough physical assessment after intubation revealed no traumatic injuries or signs of intravenous drug abuse. Blood pressure stabilised at 110/20. Pulse frequency was 95 beats per minute. Arterial blood gas was normal throughout. The patient was normothermic. She was then kept sedated and ventilated overnight in our ICU.

After being extubated the next day, she could tell us how she had intoxicated herself on lamotrigine. She was diagnosed with a bipolar disorder and, as part of her mood stabilising treatment, she was being treated with lamotrigine 400 mg 2 + 0 + 0 + 2. She also took fluoxetine 20 mg 0 + 0 + 0 + 3, carbamazepine 200 mg 1 + 0 + 1 + 1, thiamine 100 mg 1 × 1, and finally a vitamin B complex supplement. Serum lamotrigine concentration was 191.9 micromol/L. The therapeutic reference area is 10–60 micromol/L. Neither our patient's history nor our toxicology screen suggested coingestion with any other substance, legal or otherwise.

## 3. Discussion

Lamotrigine is a membrane stabilising and inhibitory drug that acts by blocking presynaptic sodium channels. In doing so, lamotrigine blocks release of the excitatory neurotransmitter glutamate. Another proposed mechanism, suggested by animal studies, is presynaptic blockage of potassium channels, which causes an increase in the release of GABA, an inhibitory transmitter [2]. Through its membrane stabilising and inhibitory effects, lamotrigine has, since 1991, been used as an antiepileptic drug and is widely used for treating partial and generalised seizures. Lamotrigine has also found a wider indication as a mood stabiliser, particularly for treating bipolar I disorder and bipolar depression [3].

Our patient had tachycardia, seizures, ataxia, and nystagmus. This clinical presentation is consistent with lamotrigine overdose. In a 2004 review of lamotrigine toxicity, Lofton and Klein-Schwartz reviewed 493 single-substance toxic exposures to lamotrigine. The majority of patients (52.1%) exposed to lamotrigine had no toxic clinical effects. The most common clinical effects reported in the minority of patients were drowsiness, vomiting, nausea, dizziness, and tachycardia. Serious clinical effects were rare and included coma, seizures, respiratory depression, intraventricular conduction delay, nystagmus, and ataxia [4].

Ketamine, used as a sedative and anaesthetic agent, produces dissociative anaesthesia, a unique state characterised by catalepsy, analgesia, and amnesia [5]. In Scandinavian emergency departments and in prehospital care, ketamine is often the preferred agent when patients are unstable or their diagnosis is unclear. Ketamine is an antagonist of glutamate's ligand action on the excitatory N-methyl-D-aspartate (NMDA) receptor [6]. However, ketamine's exact mechanisms of action remain unknown. An expanding body of research implicates complicated interaction with other receptor systems, for example, opioid receptors [7]. In addition, ketamine displays local anaesthetic action, possibly by blocking sodium channels [8]. Ketamine also interacts with muscarinic acetylcholine receptors, most likely by acting as an antagonist [9]. Finally, most relevant in the context of our case report, the current research suggests ketamine's neuropsychiatric effects and dissociative anaesthesia may be mediated via increased glutamate release outside the NMDA pathway. Ketamine, while directly antagonising glutamate's effect on the NMDA receptor, actually increases glutamatergic transmission through pathways not involving the NMDA receptor [10, 11].

If ketamine's neuropsychiatric effects, in part, are mediated by augmented glutamatergic neurotransmission, then agents lowering glutamate transmission, like lamotrigine, could attenuate these neuropsychiatric effects. Experimental studies have demonstrated how lamotrigine actually inhibits the dissociative effects of ketamine. The first study, by Anand et al., tested the hypothesis that ketamine produces its dissociative effects through increased glutamate. Sixteen volunteers were randomized into oral pretreatment with either 300 mg lamotrigine or placebo. The subjects were then injected with an intravenous ketamine bolus of 0.26 mg/kg followed by an infusion of 0.65 mg/kg/h. The study was able to demonstrate how the increase in psychosis score on the Brief Psychiatric Rating Scale, caused by ketamine, was attenuated in the lamotrigine group, but not in the placebo group. Interestingly, compatible with our patient's symptoms, Anand et al. also noted how ketamine's mood-elevating effects at the same time were enhanced by lamotrigine [12].

Then, in 2008, Deakin et al. demonstrated how ketamine changed regional blood flow in a pharmacological magnetic resonance imaging (phMRI) study. Pretreatment with lamotrigine prevented many of these changes from occurring [13]. Recently, phMRI was also conducted in a randomized, placebo-controlled crossover design in healthy volunteers. The study demonstrated the ketamine phMRI response and its attenuation with both lamotrigine and risperidone [14].

## 4. Conclusion

Our patient was admitted to our ED after ingesting a toxic dose of lamotrigine, unknown at that time. In order to safely manage the patient, we opted to sedate her with ketamine. Despite being injected with a total of 250 mg ketamine, our patient displayed no signs of dissociative anaesthesia. Ketamine's dissociative effects are due to increased glutamate release and action outside the NMDA pathway. Lamotrigine is an inhibitor of glutamate release and attenuates ketamine's dissociative effects. This is important knowledge for anaesthesiologists in the emergency room, where ketamine is often considered the safest anaesthetic to administer to unstable patients.
